# *Thetha Nami*: participatory development of a peer-navigator intervention to deliver biosocial HIV prevention for adolescents and youth in rural South Africa

**DOI:** 10.1186/s12889-021-11399-z

**Published:** 2021-07-13

**Authors:** Maryam Shahmanesh, Nonhlanhla Okesola, Natsayi Chimbindi, Thembelihle Zuma, Sakhile Mdluli, Nondumiso Mthiyane, Oluwafemi Adeagbo, Jaco Dreyer, Carina Herbst, Nuala McGrath, Guy Harling, Lorraine Sherr, Janet Seeley

**Affiliations:** 1 Africa Health Research Institute, Durban, KwaZulu-Natal South Africa; 2 Institute for Global Health, University College London, Capper Street, London, WC1E 6JB UK; 3 University of KwaZulu-Natal, Durban, KwaZulu-Natal South Africa; 4 University of Johannesburg, Johannesburg, Gauteng South Africa; 5 University of Southampton, Southampton, UK; 6 MRC/Wits Rural Public Health & Health Transitions Research Unit (Agincourt) University of the Witwatersrand, Johannesburg, Gauteng South Africa; 7 Department of Epidemiology & Harvard Center for Population and Development Studies, Harvard T.H. Chan School of Public Health, Boston, USA; 8 London School of Hygiene and Tropical Medicine, London, UK

**Keywords:** HIV, Health promotion, Peer-led, Community-based participatory research, South Africa, Pre-exposure prophylaxis, Sexual health, Adolescents, Young people, Social capital

## Abstract

**Background:**

Despite effective biomedical tools, HIV remains the largest cause of morbidity/mortality in South Africa – especially among adolescents and young people. We used community-based participatory research (CBPR), informed by principles of social justice, to develop a peer-led biosocial intervention for HIV prevention in KwaZulu-Natal (KZN).

**Methods:**

Between March 2018 and September 2019 we used CBPR to iteratively co-create and contextually adap*t* a biosocial peer-led intervention to support HIV prevention. Men and women aged 18–30 years were selected by community leaders of 21 intervention implementation areas (*izigodi*) and underwent 20 weeks of training as peer-navigators. We synthesised quantitative and qualitative data collected during a 2016–2018 study into 17 vignettes illustrating the local drivers of HIV. During three participatory intervention development workshops and community mapping sessions, the peer-navigators critically engaged with vignettes, brainstormed solutions and mapped the components to their own izigodi. The intervention components were plotted to a Theory of Change which, following a six-month pilot and process evaluation, the peer-navigators refined. The intervention will be evaluated in a randomised controlled trial (NCT04532307).

**Results:**

Following written and oral assessments, 57 of the 108 initially selected participated in two workshops to discuss the vignettes and co-create the *Thetha Nami* (`talk to me’). The intervention included ***peer-led health promotion*** to improve self-efficacy and demand for HIV prevention, ***referrals to social and educational resources****,* and ***aaccessible youth-friendly clinical services*** to improve uptake of HIV prevention. During the pilot the peer-navigators approached 6871 young people, of whom 6141 (89%) accepted health promotion and 438 were linked to care. During semi-structured interviews peer-navigators described the appeal of providing sexual health information to peers of a similar age and background but wanted to provide more than just “*onward referral*”. In the third participatory workshop 54 peer-navigators refined the *Thetha Nami* intervention to add three components: ***structured assessment tool*** to tailor health promotion and referrals, ***safe spaces and community advocacy*** to create an enabling environment, and ***peer-mentorship and navigation*** of resources to improve retention in HIV prevention.

**Conclusion:**

Local youth were able to use evidence to develop a contextually adapted peer-led intervention to deliver biosocial HIV prevention.

**Supplementary Information:**

The online version contains supplementary material available at 10.1186/s12889-021-11399-z.

## Background

South Africa (SA) has an estimated 7.7 million people living with HIV – the highest number globally - and HIV remains the leading cause of death. HIV incidence has remained stubbornly high, especially in KwaZulu-Natal (KZN) where we have shown an annual incidence of 8% amongst females aged 20–24 and 4% in males aged 25–29 [1]. This is despite highly efficacious and cost-effective HIV prevention tools. These include HIV point-of-care tests (POCT) and self-tests; the use of daily oral tenofovir/emtricitabine for Pre-Exposure Prophylaxis (PrEP), which can reduce the risk of acquiring HIV by up to 90%; voluntary medical male circumcision that reduces the risk for men of acquiring HIV by 60%; and HIV treatment with antiretroviral therapy (ART) that reduces mortality and eliminates onward transmission of HIV to sexual partners [2–4].

This failure to arrest the HIV epidemic is partly a result of the disparity between the vulnerability of subpopulations to HIV and their use of HIV prevention tools. Adolescents and youth in South Africa are one such subpopulation where the intersecting socially determined inequalities, namely youth, gender and poverty, have prevented them from effectively engaging with bio-behavioural HIV prevention [5, 6]. Concern about young people’s vulnerability to HIV-infection led to the launch of initiatives, such as the DREAMS (determined, resilient, empowered, mentored and safe) partnership that combines social and behavioural interventions to reduce adolescent and youth vulnerability [7]. However, such initiatives have struggled to accelerate the declines in HIV incidence [8].

There is growing evidence of the effectiveness of community-based HIV care. A meta-analysis found that community health worker HIV care delivery significantly improved HIV viral suppression, which also reduces sexual transmission (pooled OR: 1.40 95% CI 1.06–1.86) [9]. The DOART trial showed that community-based care was superior to facility-based HIV treatment in supressing HIV viral load [10]. Community-based approaches - particularly when integrated with psychosocial care - can foster social networks, mobilise HIV competent communities and stimulate progress towards the sustainable development goals [11–17]. This is particularly important for adolescents [18]. This was shown in a peer-led intervention for adolescents living with HIV in Zimbabwe, the first study to improve adolescent HIV viral suppression [12, 19, 20].

Peer-to-peer approaches are advocated as sustainable ways to harness social learning and adapt it to the social context [21]. This approach is particularly powerful when it evolves into collective action that drives social movement for change [21–25], or engages with pedagogical theories to support socially embedded co-production of knowledge [26]. A systematic review identified 12 studies that examined peer-based interventions with young people and found improvements in knowledge, sexual behaviour, and condom use [27] and evidence for peer-led interventions to support HIV prevention is emerging [18, 28–30]. However, there remains a need for rigorous evaluation of the effectiveness of peer-led interventions, with well-understood components, on health outcomes [25, 26, 31].

### Conceptual framework for the intervention development

We used a community-based participatory research (CBPR) to develop the intervention. Our approach was informed by Bourdieu’s (1986) critical and power-focused understanding of social capital as applied to global public health by Campbell [32]. CBPR creates a transformative space for a community to engage in critical dialogues that generate context-relevant knowledge and action [33–35]. This process of social learning and solidarity encourages bonding social capital [16, 32]. However, as Campbell et al. have shown in their analysis of successful social movements, bonding social capital is not sufficient for health related change [22, 36]. They argue that the networks of solidarity should mobilise communities (bridging social capital) and link to institutions and champions (linking social capital) to create HIV competent communities and collective action for health enabling social change [16, 32]. This approach has not been consistently applied with adolescents and youth [37–39].

We hypothesise that social justice informed CBPR (rooted in the principles of equity, access, participation and rights) will foster youth-led transformative action to tackle the HIV epidemic in sub-Saharan Africa. In this paper we interrogate how young people - using social spaces for critical dialogue to foster solidarity and action (bonding social capital), seeking local partnerships (bridging social capital), and mobilising resources (linking social capital) - contextually adapted biosocial HIV prevention intervention in rural KwaZulu-Natal (KZN), South Africa. We examine the process of co-creation of intervention, in accordance with these theoretical components. The effectiveness of this peer-led biosocial HIV prevention intervention, *Thetha Nami* (“*talk to me”* in isiZulu) will be evaluated in a future randomised controlled trial (NCT04532307).

## Methods

Between March 2018 and September 2019, we used CBPR to iteratively co-create and contextually adapt a biosocial intervention to be delivered by community-based peer-navigators. We used a logic model to show how and why the components of the intervention strengthened social capital and improved demand, uptake and retention along the HIV prevention cascade (Theory of Change).

### Setting

This study was conducted by the Africa Health Research Institute (AHRI) in the uMkhanyakude district of KZN. The study area of ~ 500 km^2^ with a population of ~ 17,000 16–29 year-olds was the site of the scale-up of structural and behavioural interventions through the DREAMS partnership between 2016 and 2018 [7]. The study area is mostly rural and poorer than most other parts of South Africa, with high levels of unemployment and a high burden of HIV [1, 40, 41].

### Ethics

Ethics approval was received by the Biomedical Research Ethics Committee of the University of KwaZulu-Natal, South Africa (BFC515/18) and of University College London, United Kingdom (5672/002). Peer Navigators underwent a process of informed consent prior to interviews and group discussion. It was made clear to the Peer Navigators that participation in all aspects of data collection was voluntary and they were able to withdraw at any time. To ensure they were confident that withdrawal would not impact on their employment, their line management was distinct from the team who supported CBPR. The process evaluation team, who collected data, were also independent from CBPR team.

### Patient public engagement

The study was presented to the community advisory board, peer-navigators and the District Department of Health before submission to Institutional Review Boards. CBPR was used to provide youth input throughout the process and into the final peer-navigator interventions.

### Role of the researchers

One team of four social science researchers were led by TZ (an *isiZulu* speaking postdoctoral research psychologist) to support CBPR. A second team of four social science researchers were led by OA (a postdoctoral social scientist) to conduct the process evaluation. All researchers were *isiZulu* speaking and had lived and worked in the community for over 5 years. They had been trained in group facilitation, qualitative data collection, transcription, translation and coding. The day to day research was overseen by multidisciplinary research management group which included the principal investigator (MS), the study programme manager (NC), study coordinator (CH), professional nurse (NO), data manager (JC), and senior social scientists (TZ and OA). A multidisciplinary team of investigators led by MS, LS (psychology), JS (social anthropology), GH (social networks), and NM2 (social epidemiology), provided iterative input to the Theory of Change.

### Participatory intervention development

**Fig. 1:**
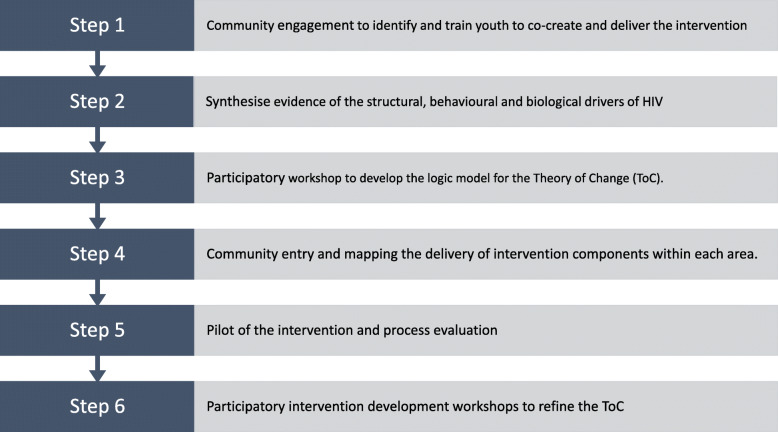
Steps to develop Thetha Nami participatory peer-led biosocial intervention

The process followed six steps in which through listening and responding to young people a biosocial intervention iteratively emerged (Fig. 1).

### Step 1: community engagement to identify and train youth to co-create and deliver the intervention

With the support of the AHRI community advisory board and public engagement unit, local traditional and municipal leadership from the 21 administrative areas (*izigodi*) were asked to identify 4–5 young men and women living in their area for training to support youth in their areas. The criteria for selection, developed with the AHRI public engagement unit, were that the youth had to: be aged 18–30; have completed high school and matriculated; be actively engaged in their communities; and be considered opinion leaders [42]. We strongly encouraged leadership to select both young men and women. Participants underwent training which covered, youth development, HIV and sexual health information, HIV counselling and testing course (accredited by the South African Health and Welfare Sector Education and Training Authority), confidentiality, ethics, and research methods. The training had originally been planned for 16 weeks (supplementary file 1). Progress was evaluated using written and oral assessments and to ensure that there was at least one pair of peer navigator residing in each of the designated areas. This resulted in 57 successfully trained peer-navigators being employed for 24 h work per week to co-create and implement the intervention in their areas.

### Step 2: synthesise the existing evidence of structural, behavioural and biological drivers of HIV

We used quantitative and qualitative data from the evaluation of the local scale-up of structural and behavioural interventions through the DREAMS partnership between 2016 and 2018 to summarise the structural, behavioural and biological drivers of HIV, and of engagement with the HIV prevention cascade [7, 8, 43]. The evaluation data arose from: (i) surveys with a representative sample of 13–35-year-old-males and females (*n* = 4918); (ii) rapid ethnographic community mapping (n = 4); (iii) provider and user interviews (*n* = 22 and *n* = 58 respectively); and (iv) group discussions (*n* = 29). Detailed methods and findings of the contextual factors that drive risk and create barriers to effective HIV testing, care and prevention have been previously reported [1, 6, 40, 43–49]. For the purposes of the intervention development the findings were summarised and converted into vignettes, case-studies and simple infographics by a team of social scientists, statisticians and clinicians who had been engaged in the data collection and peer-navigator training (supplementary file 2).

### Step 3: participatory workshop to develop the logic model for the theory of change (ToC)

**Fig. 2:**
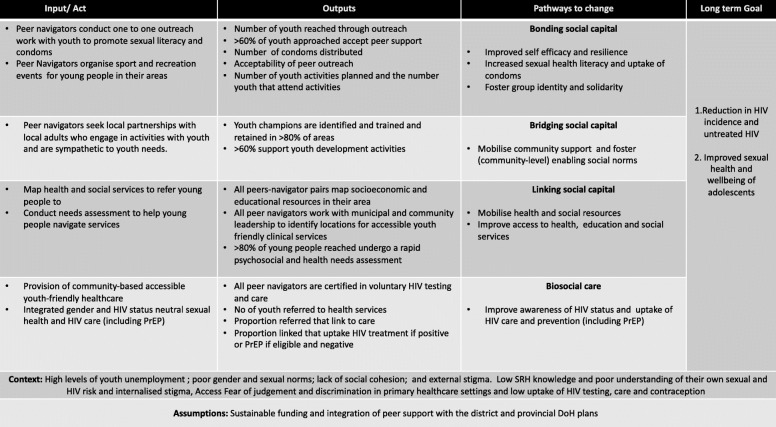
Thetha Nami peer-led biosocial HIV prevention intervention: Theory of Change (ToC) Phase one

During two full day participatory workshops conducted in isiZulu in the research institute premises, participants were divided into seven mixed gender small groups of 6–11 individuals according to the proximity of their areas of residence. Each group was moderated by a social science researcher (see above). Participants were instructed to emerge from the group work with interventions they could implement themselves. They could mobilise local resources and biomedical HIV prevention tools provided by the study. They used the vignettes as a vehicle to critically engage with and brainstorm practical approaches (through a medium of their choosing e.g. role play, pictures, or story telling) to mitigate the particular drivers of HIV and poor engagement with HIV care that the vignette signified. The candidate interventions were then plotted to a ToC (Fig. 2).

### Step 4: community entry and mapping the delivery of intervention components to each area (*izigodi*)

The peer navigators were divided into three groups who each worked closely with one social scientist. They were supported by the research management team (above) who met weekly. Each group: i) physically mapped the health, education and social services within their own communities. This included the components of the primary health care re-engineering in their areas, i.e. the primary healthcare staff, community care givers, schools and school health teams; ii) mapped the places that young people gather; iii) identified potential adult youth champions to support them; iv) piloted the structured needs assessment tools (supplementary file 3); v) implemented the health promotion intervention under the observation of their supervisors; and vi) identified places for youth-friendly mobile healthcare services (***Isisekelo Sempilo*** **(‘*Foundation of Life****”* in *isiZulu***).** They then came together in a second workshop to harmonize approaches in each *izigodi*.

### Step 5: pilot of the intervention and process evaluation

The intervention was piloted across the 21 *izigodi* with some of the larger or more densely populated areas having more than one pair of peer navigators. We conducted a process evaluation of the pilot using a combination of programme data, data collected from the training and supervision and semi-structured interviews with a purposive sample of peer-navigators. We collated data from anonymised programme data records of the peer navigators’ daily reporting of their outreach activities. This included date and time, age, gender, area of recruitment, the peer navigator ID and the service provided by the peer navigator. This was supplemented with reflective field notes and notes taken by the social science supervisors from bi-weekly training sessions, and weekly supervisory debriefings to assess the feasibility and acceptability of the intervention. Semi-structured interviews were conducted between April and August 2019 with 34 purposively selected Thetha Nami peer navigators aged 20–30 years (24 female and 10 male), to represent the different areas and include both genders [50]. The interviews were conducted in *IsiZulu*, audio-recorded, transcribed and later translated to English. Participants’ views were explored about the acceptability and feasibility of *Thetha Nami* and interviews lasted between 30 to 60 min. Interview transcripts were managed and coded using NVIVO software and data were analysed thematically following an interpretivist approach [50].

### Step 6: participatory intervention development workshops to refine the intervention and ToC

**Fig. 3:**
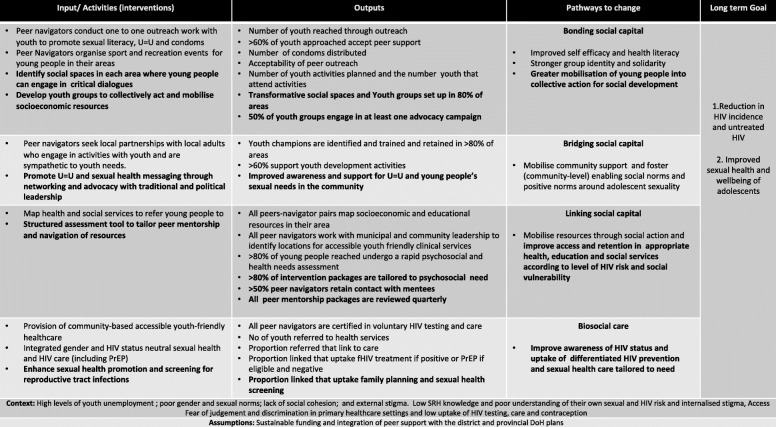
Thetha Nami peer-led biosocial HIV prevention intervention: Theory of Change (ToC) Phase two

We conducted a third participatory workshop, facilitated similarly to the workshop described in step 3, to discuss and rank the challenges to implementation and refine the intervention and ToC (Fig. 3). Peer navigators were presented with the process evaluation findings and asked to reflect on their own experiences. They were divided into three moderated groups and asked to identify the challenges and rank their top three. They then deconstructed each challenge, described what its signified and brainstormed potential solutions. These were then presented pictorially back to the whole group.

The effectiveness of *Thetha Nami* on HIV outcomes will be tested in a randomised controlled trial with process evaluation (NCT04532307).

## Results

### Step 1: community engagement to identify and train youth to co-create and deliver the intervention

Between March 2018 and June 2018, the traditional and municipal leadership identified 42 men and 66 women as potential peer-navigators. All 21 areas were able to identify around five youth who fitted the inclusion criteria and agreed to participate. The success of recruitment was partly because of the enthusiasm the community leaders expressed for a programme that provided training and employment to youth.

Between June and September 2018 participants underwent a 20-week training programme (3 days a week). The training had originally been planned for 16 weeks, but due to the low levels of sexual and reproductive health knowledge was extended by 4 weeks. Fifty-seven peer-navigators were selected to implement the intervention. Although the plan had been to select male-female pairs, only 13 of the 57 people selected by the assessments and interviews were men. Retention was good with 53 (92%) still working after 1 year, of which 12 (23%) were men, and 19 (36%) aged 18–24. All four who left the programme took up full time work or education.

### Step 2: synthesise evidence of the structural, behavioural and biological drivers of HIV and poor engagement

Table 1 summarises the key drivers of risk and barriers to uptake of HIV care and prevention drawn from the survey data (*n* = 4918) and studies conducted between 2016 and 2018 in our setting. These data formed the basis of the development of 17 vignettes, which took the form of narratives, pictures, and case-studies that had cultural resonance and were rooted in the context (supplementary file 2).

**Table 1:** Drivers of risk and barriers to effective uptake of multi-level HIV prevention for adolescents and young adults in rural KZN drawn from survey data (*n* = 4918) and studies conducted 2016–2018 [1, 6, 8, 37, 40, 43–49, 51–56]

Unmet need and challenges	Consequences of unmet need
Social vulnerability of youth	• High unemployment (85% of school-leavers are unemployed)
	• Migration (30% moved location in past year)
	• Transactional sex (13% in past year)
Sexual health needs	• 20% of women and 10% of men had a curable STI.
	• 75% of these reporting no symptoms,
	• 40% of the women had bacterial vaginosis.
	• Home-based self-sampling and treatment for STIs was acceptable and desirable to young people
	• Teenage pregnancy levels are persistently high, with an annual incidence of teenage pregnancy of 6.4% (5.7–8.6) (unpublished data)
	• The majority of young women 15–24 start contraception after their first pregnancy.
	• Poor sexual health and knowledge despite the importance of fertility
Unmet mental health needs	• High levels of common mental disorders (CMD) which increase with age (rising to 32% of those aged 20–22).
Challenges to uptake of HIV prevention interventions	• Multiple service providers
	• Increasing uptake of community-based interventions (social asset building; community mobilisation and parenting programmes) over the 2 year period
	• Less success in reaching older adolescents, those out of school, and those who move
	• Young boys felt excluded – apart from Voluntary Medical Male Circumcision.
	• Limited uptake of regular HIV testing – despite over 94% knowing where to get ART and wide-spread availability of free point of care HIV testing, < 50% of 15–24-year olds tested for HIV within the previous 12 months, with pregnancy being the strongest predictor of HIV-testing in women.
	• Poor uptake of HIV care: < 20% of men aged 15–30 who tested positive linked to care.
	• Social costs (time and cost of travel, waiting times, stigma and attitude of health care providers to adolescent sexuality) of HIV testing and care outweighing any perceived benefits.
Sources of youth resilience	• Access to good sexual and reproductive health information
	• Supportive network of peers, schools, and family members
	• Social cohesion that support hope, a sense of belonging and altruism
	• Interventions that were consistent and re-enforced existing cultural and social norms

### Step 3: participatory intervention development workshops to develop the logic model for the theory of change

The first iteration of the intervention (Fig. 2) was co-created through critical dialogues with the vignettes through the medium of storytelling, pictures and role-play in the first workshop (June 2018). The approach that emerged was that area-based *Thetha Nami* peer navigators would increase the *demand and motivation for HIV prevention* by: i) peer outreach with adolescents and young people aged 15–30 to increase sexual health literacy, provide condoms, and organise group sports and recreation activities (bonding social capital). ii) mobilising the community (bridging social capital). This was achieved through identifying and training youth champions - defined as adults in the area who engage in activities that support youth and/or are sympathetic to youth needs (e.g. youth leaders, local community based organisations, teachers, community care-givers and organisers of sport and other youth recreation). *Thetha Nami* peer navigators would increase *uptake and retention* in HIV prevention through: iii) mobilising health and social resources (including those delivered locally by the Department of Health and the Department of Social development and the Integrated School Health Programme) and helping adolescents and young people navigate the resources (linking social capital); and iv) referrals to accessible youth-friendly healthcare designed by peer navigators to provide gender and HIV-status-neutral care integrated with sexual health *(Isisekilo Clinics).*

### Step 4: community entry and mapping the intervention components to the areas

Between November 2018 and February 2019 the peer navigators worked with the social scientists and professional nurses to successfully enter and map all 21 *izigodis* and conducted a second workshop to harmonize activities (November 2018). Peer navigators were introduced to all the schools in 20 areas (91%) and identified adult youth champions in 20 areas (91%). Peer navigators worked with the municipal and community leadership to identify two accessible and busy peri-urban primary health care clinics, with adolescent and youth friendly services to strengthen. They were able to identify sites for ***Isisekelo Sempilo*** youth-friendly mobile clinics in all 21 areas. The mobile and fixed clinics aligned with the KZN re-engineering primary health to deliver nurse-led pregnancy testing, family planning support, syndromic management for sexual and reproductive tract infections and if male referral, to voluntary male medical circumcision. The clinics provided HIV counselling and point of care testing, antiretroviral therapy initiation if testing positive, and HIV PrEP if testing negative and eligible according to SA national guidelines.

### Step 5: pilot of the intervention and process evaluation

The *Thetha Nami* peer navigator intervention was piloted between March 2019 and September 2019. Over 6 months, peer navigators logged reaching 6871 of the 16,473 (42%) 15–30-year-old men and women living in their areas through outreach work. 6141 (89%) accepted a rapid psychosocial and health needs assessment (supplementary file 3). Based on the assessments, the following needs were identified: referral or support for health needs, *n* = 2790; social welfare needs, *n* = 435; social vulnerability, *n* = 236; educational (skills) support need, *n* = 2330; and legal and advocacy support, *n* = 338. Peer navigators distributed 41,450 male condoms and 3000 female condoms and provided 4145 information packs and referrals to ***Isisekelo Sempilo*** **clinics, **and 438 (11%) of them attended clinic. Peer navigators organised three activities (two music festivals and one soccer tournament) attended by 156 young people (aged 15–30) in total. The events were designed to create a relaxed and non-judgemental space for youth to discuss sexual and reproductive health (sexually transmitted infections, HIV and teenage pregnancy) with peer navigators and attending clinical staff [34].

Semi-structured interviews with peer navigators highlighted three core themes (Supplementary file 4). *Comfort with dialogue theme* suggested that they found young people were comfortable to share health-related issues, mostly sexual health and condom use, that they would not have shared with adults. They appreciated the wider support (e.g., education services, CV writing). However, the *age as a barrier to respect* theme revealed that the peer-navigators were anxious that their young age was a barrier to being taken seriously by older adolescents. *Message conflict* was also encountered, when the health information they promoted, conflicted with the messages young people were receiving from their caregivers e.g., PrEP which was perceived to encourage young people to have sex. Peer navigators also felt inadequate when they could only refer participants to existing and sometimes unpopular service.

### Step 6: participatory intervention development workshops to refine the ToC

In September 2019 we conducted the third participatory workshop with 54 peer navigators. They identified their highest-ranked challenges as: *Thetha Nami* not being valued by youth; young people being “*lazy*” to engage with the ***Isisekelo Sempilo*** clinics; and HIV stigma. First they deconstructed what these challenges signified**:**

*Thetha Nami being seen as “useless by youth”.* Peer navigators identified this as partly due to the logistic barriers they faced in delivering *Thetha Nami*: small age differences particularly with older adolescents; lack of safe spaces for youth to gather; and size of the rural areas with poor transport links. However, on reflection they identified this “*uselessness*” as signifying gaps in the services they had at their disposal to offer youth. In particular they were concerned that they lacked concrete social welfare and employability services to refer participants to and were reliant on referring to existing and stretched government services.

Youth are *“lazy”* to engage with HIV testing and biomedical prevention and care. *“Laziness”* emerged as meaning *“scared”.* They described this overwhelming fear amongst youth to test for HIV because they are “*scared”* to know their HIV status, “*scared”* to find out they are HIV seropositive, and then “*scared”* to disclose to their families. When reflecting on their frustration at the apparent failure of their positive messaging and provision of adolescent- friendly convenient services to mitigate this “*scaredness*” they identified two further challenges: (i) the messaging around HIV treatment rendering individual uninfectious (“Undetectable = Untransmissible” or “U=U”) and PrEP (to reduce HIV acquisition) were new concepts to both for the peer navigators and community members. This led to resistance from young people that made it hard for them to promote these novel biomedical ideas and (ii) the resistance from caregivers and elders to these novel interventions that were perceived to *“encourage”* adolescent sexual activity.

*HIV Stigma* was seen as a result of fear which they divided into internal fear, i.e., things young people feared would happen to them if they tested HIV-positive; and external fear, i.e., things young people feared others would say or do to them if they were known to have HIV or be at risk of HIV. Internal stigma were seen to be fueled by some of the persistent beliefs surrounding HIV. For example, that HIV treatment is arduous (e.g., the need to have food with ART, having to take ART the same time every day, not being able to mix alcohol with ART), and the anticipated negative psychological effect of knowing their status, described as “*killing”* them. External HIV stigma was fueled by others seeing them as **“***dirty, immoral and infectious***”** and not worthy of being in a “*healthy sexual relationship”.*

The groups then brainstormed adaptations to the *Thetha Nami* intervention to tackle these challenges . These were mapped to a revised ToC (Fig. 3):

i. Adapt the tablet-based psychosocial and health needs assessment tools used with young people to provide a risk differentiated and individualized health promotion plan for peer-mentorship (supplementary file 5).
ii. Identify social spaces in each area that the peer navigators work where young people can gather and receive non-judgmental sexual and reproductive health information, socialize safely and engage in critical dialogues [34]
iii. Develop youth groups to collectively act and mobilise local networks and resources to improve their socioeconomic conditions e.g., skills training, apprenticeships, investment and saving schemes, and starting small businesses [34].
iv. Enhance sexual health promotion with screening for reproductive tract infections
v. Advocate for “U=U” and young people’s sexual health needs with youth champions, parents/carers, and local political and traditional leadership.

### *Thetha Nami* peer-led biosocial intervention

The final co-created *Thetha Nami* intervention that will be evaluated consists of area-based peer navigators working to increase the *demand and motivation for HIV prevention* by i) developing youth groups and transformative social spaces for critical dialogue and solidarity (bonding social capital); and ii) creating an enabling environment for youth through community-wide health promotion; networking with youth champions and advocacy with the local political institutions (bridging social capital); and iii) supporting (individual-level) differentiated HIV prevention that is tailored to need and pivots around sexual health literacy and mentorship to support self-efficacy. Peer navigators would increase *uptake and retention* in HIV prevention and care through social action to mobilise resources (linking social capital): i) mapping the health, education and social services within their own communities; ii) provision of accessible youth-friendly healthcare that provides HIV status neutral care within an enhanced sexual health service; and iii) provide ongoing tailored peer-support and lay counselling of young people to retain them in care.

## Discussion

Using evidence of what worked and did not work for young people like them, local youth were able to design and develop a peer-led intervention, underpinned by the principles of social justice, to deliver a biosocial HIV prevention. The use of evidence derived vignettes as vehicle to support critical dialogues and the mobilisation of wider community support from the start provided a platform for young people to engage creatively with the evidence and contextually adapt effective interventions to a rural and deprived setting. Moreover, by iteratively adapting the intervention to the contextual challenges identified through the process of intervention development, the *Thetha Nami* peers reached more than one in three of all young people residing in their area over a six-month period.

*Thetha Nami* explicitly tackled the challenges young people found in identifying themselves as candidates for the biomedical HIV care and prevention [46] through two mechanisms: First by emphasizing an HIV status neutral health promotion message that pivoted around sexual health. Second by removing structural barriers to accessing care by tackling community norms around adolescent sexuality and delivering accessible and youth friendly clinical services. Thus *Thetha Nami* echoes the calls for HIV status neutral approaches in high income settings [57] and to integrate PrEP with sexual health services [58]. CBPR enabled the peer-navigators to adapt the biosocial intervention to their own rural setting. Moreover, by using the needs assessment tool to tailor health promotion they can differentiate support at an individual level, with those with greater need receiving the greatest amount of peer support [19, 59].

Through the participatory process the peer navigators identified the need for community wide interventions, such as safe spaces and peer mentorship, to create a protective environment. This resonates with earlier findings of HIV competent communities [16] and is theoretically aligned with the protection-risk framework [60, 61]. The emphasis on youth leadership and bonding, bridging and linking social capital as key ingredients set *Thetha Nami* apart from otherwise similar combination prevention interventions for adolescent girls and young women such as DREAMS [7]. Indeed *Thetha Nami* bears closer resemblance to the combination interventions that emerged from sex worker mobilization and collectivization and the ‘push from below’ embodied in the Treatment Action Campaign in South Africa [17, 22, 24, 32, 36, 59, 62]..

In this area of high youth unemployment, the peer navigator intervention was seen as beneficial not only to the community but also the peer navigators themselves by providing training, work and income. The benefits of participating in peer mentorship programmes on the peers themselves has been described in other programmes [12, 13, 19, 21, 25, 58]. *Thetha Nami* like some other programmes, if integrated with the wider health and social care system, can be a vehicle to accelerate sustainable development goals amongst adolescents [11].

Our study results show that internalised and externalised HIV stigma and social norms around adolescent and youth sexuality remain a huge challenge to delivering HIV prevention, even in these high HIV burden rural settings [63–65]. Young people involved in this research brought a nuanced understanding of the intersection between poverty and lack of opportunity, and the gender and intergenerational power imbalances to the intervention development process [66, 67]. The solution they proposed is a youth-led intervention to organise and mobilise other young people and the wider community to challenge social norms and lack of opportunity, and access healthcare within their own locality.

## Conclusion

We found that CBPR that used evidence derived vignettes to support critical dialogues was a powerful way for local youth to contextually adapt biosocial HIV prevention and deliver it through peer-to-peer approaches to a third of all young people in their locality. Principles of social justice emerged as integral to tackling the intergenerational and socioeconomic power imbalances that underpin the HIV epidemic in this rural community. Thus, youth leadership and bonding, bridging and linking social capital were key ingredients to *Thetha Nami* peer-to-peer delivery of biosocial HIV prevention.

With increasing need for long-term prevention within universal health care, effective and efficient models of community delivery of care for the growing number of young people is important, not just for HIV, but wider adolescent health [68]. We show that *Thetha Nami* is an acceptable and feasible way to deliver differentiated biosocial HIV prevention that is tailored to need to large numbers of young people in a rural and deprived setting of KZN. Furthermore, it is scalable and sustainable through the community caregiver programs in southern Africa. The effectiveness and cost effectiveness of this peer-led biosocial intervention to reduce HIV-related mortality and morbidity will be tested in a randomized controlled trial (NCT04532307) that will report in 2022.

## Supplementary Information

**Additional file 1 ***Thetha Nami* Peer Navigator Training Programme.

**Additional file 2 ***Thetha Nami* Participatory Mapping Workshop Tools.

**Additional file 3 ***Thetha Nami* Peer Navigator Monitoring Tool.

**Additional file 4.** Peer Navigator indepth interview topic guide.

**Additional file 5 ***Thetha Nami* Peer Mentorship Tool.

## Data Availability

The datasets generated and/or analysed during the current study are available in the AHRI repository. Data can be requested from the AHRI External Data Repository (https://data.ahri.org). Peer navigator pilot process data is in the Thetha Nami Information dataset in the “Get Microdata” tab of (https://data.ahri.org/index.php/catalog/978). Data from which the vignettes were developed was the DREAMS Impact Evaluation Project–Nested Cohort–Standardized and combined dataset for all 3 Rounds - https://data.ahri.org/index.php/catalog/977. The qualitative datasets used and/or analysed during the current study are available from the corresponding author on to access on a secure database to researchers upon reasonable request.

## Abbreviations

AHRI: Africa Health Research Institute
ART: HIV Antiretroviral Therapy
CBPR: Community-Based Participatory Research
DREAMS: Determined, Resilient, Empowered, Mentored and Safe
KZN: KwaZulu-Natal
POCT: HIV Point of Care Test
PrEP: HIV Pre- Exposure Prophylaxis
SA: South Africa
ToC: Theory of Change
U=U: Undetectable = Uninfectious
