# Supplementary material for: Thetha Nami: participatory development of a peer-navigator intervention to deliver biosocial HIV prevention for adolescents and youth in rural South Africa
Source: BMC Public Health. 2021 Jul 13;21:1393. doi: 10.1186/s12889-021-11399-z (PMC8278686; doi:10.1186/s12889-021-11399-z)
Supplement: Supplementary file 1 — Additional file 1 Thetha Nami Peer Navigator Training Programme. [file 12889_2021_11399_MOESM1_ESM.pdf]

PEER NAVIGATORS TRAINING PROGRAMME AND LEARNING OBJECTIVES		
**TITLE**	**DURATION**	**FACILITATOR**
**INTRODUCTION-** getting to know each other	45min	Nonhlanhla
**BASIC HEALTH AND SAFETY LEARNING OBJECTIVES**		Marlise
To know the expected behaviour while at AHRI premises<br>Assembly points during of emergency	1hour	Marlise/Nonhlanhla
**AHRI SOMKHELE SITE BUILDING TOUR**		
To be able to know where different departments are<br>Able to access the building	30mins	Nonhlanhla/Marlise
**HIV update and myth buster**	3hours	Londiwe/Hlolisile
Able to know HIV transmission<br>Multilevel HIV prevention<br>Able to know correct condom use<br>Able to know meaning of VL suppression<br>Why VL vs CD4<br>Able to know if HIV is curable		
**AHRI OVERVIEW OF PIP AND POPC (Vukuzazi)**	1hour	Thobeka and Resign
Able to explain, in simple terms, what Africa Health Research Institute (AHRI) does<br>Able to explain, in broad terms, what the Population Intervention Platform (PIP) aims to contribute<br>Able to list the main ways in which PIP differs from the former DSS, from a field work perspective] SHORT LIFE L.O.<br>Able to explain, in broad terms, the contribution of fieldwork to the PIP<br>Able to explain, in simple terms, that additional research modules will become part of PIP<br><br>Able to explain, in simple terms, why pilot studies are conducted<br>Able to explain, in simple terms, why randomisation is important and how it works.<br>Able to explain, as though to a participant, examples of good things that AHRI research has brought to the local population<br>Able to list major sources of funding for PIP		
**AHRI DIFFERENT STUDY INTRODUCTION**	1hour	Nonhlanhla

Able to understand each project that belong to AHRI	30mins	Nonhlanhla/Sithembile
**HOME ENTRY**	1hour	Noma/Ncengani
Able to know the home entry policy		
Able to know the study area, tribal authority and the geographic area		
Able to know and demonstrate home entry steps		
Able to know what is done and not done when one is at the homestead		
**FINAL Training for 45-55 remaining peer navigators**		
**GOOD CLINICAL PRACTICE- confirmed 28 and 29 June 2018**	2days	Sphephelo
**CHILD PROTECTION**	1day	Fikile
**Right and the law**		
Able to outline in simple terms key legal rights and responsibilities to support child protection in South Africa		
Able to give brief definitions of both abuse and child exploitation		
Able to describe the ways in which consent processes differ for under 18s- in comparison with adult		
Able to outline right to confidentiality of all children		
**Correct Age**		
Able to explain that a person’s incorrect age may sometimes be given (e.g. error; ‘round up ‘near birthdays)		
Able to give reason why knowing the correct age of a participant is essential to participants and to AHRI for ethical and legal reasons.		
Able to consistently and correctly calculate age from date of birth		
Able to define correctly the lower limit of age for inclusion as a participant in the research modules in which you are working		
**AHRI Child Protection Procedures**		
Confidentiality and children / young people		
**Able to demonstrate a consistently empathic, calm, no judgemental and professional approach with young people**		
Able to demonstrate active listening skills when appropriate, when working with young people		
Able to respond appropriately response to a young person who asks for advice about sexual health		
Able to discuss, as though to a participant, sexual health promotion including<br>• Condom use,		

• Voluntary male medical circumcision (VMMC)<br>• PreP<br>• A short explanation of commonly available methods of contraception		
Able to demonstrate appropriate reassurance and support to a young person when indicated		
Able to demonstrate appropriate advice-giving to young person to indicate to them the option for support or disclosure		
Able to demonstrate familiarity with referral resource that lists options for urgent and non-urgent sexual health care, and describe where to obtain a copy at short notice		
Able to demonstrate the AHRI list of services, as though to a participant, in a way appropriate to the problem in hand		
Able to perform simple assessment of possible child protection incident		
Able to explain in simpler terms how untreated HIV affects health over time	3hours	Nonhlanhla/Clinical team
Able to explain the ways in which a) HIV is transmitted and how transmission can be prevented, or b) risk of transmission reduced		
Able to explain in simple terms the three UNAID's '90s' are (can express as 9 out of 10people) for HIV		
Able to explain in simple terms what the benefits for the communities and individuals of achieving the 90/90/90		
Able to outline some of the commoner reason that people who know they have HIV, are slow to link to care-or fail to link		
Able to list to the participant the advantages of knowing their HIV status		
Able to explain to a participant the advantages of regularly taking of ARV's to individual and community		
Able to explain in simple terms, as though to the participant, what is meant by term viral load (including suppressed and high results)		
Able, in simple terms, describe what needs to be done by individual to maintain a fully suppressed viral load		
Able to discuss, a though to a participant, sexual health promotion including condom use, VMMC and knowing own HIV status and that of a partner		
Able to demonstrate the correct and appropriate approach to asking individual questions and give an example when it is useful		
**GEOGRAPHIC INFORMATION SYSTEM**	2hours	Sabelo
**Mapping and GPS**		

Able to describe, with help of a map, the boundaries of the PIPSA, including the north and south parts		
Able to describe or name important landmarks at key points of the PIP boundary		
Able to scan documents correctly and reliable use of barcodes		
**ADVERSE EVENTS**	2hours	Gugu
Able to know in simple terms the meaning of AE		
Able to know how to report an AE		
Able to know what to report as an AE		
**THE OVERVIEW OF STAR PROJECT**	2hours	Carina
Able to explain in simple terms the STAR project to the community		
Able to give information about HIV self-testing screening		
Able to demonstrate how to use self- testing kit		
**CLINICAL GOVERNANCE**	2hours	Philippa
Able to understand and explain in simple terms what clinical governance means		
**LINKAGE TO CARE**	1hour	Sanah/Siphephelo
Learning objectives		
**CORRECT IDENTIFICATION OF PARTICIPANTS**	2hours	Nonhlanhla
**HANDLING OF TABLET**	1hour	Jaco
Able to know how to handle and use a tablet		
Able to trouble shoot when using the tablet		
**HANDLING OF ELECTRONIC DATA**	1hour	Jaco
Able to know what RedCap is		
Able to know how to use RedCap		
Able to know the confidentiality that goes with electronic data and how it is kept		
**PROTOCOL**	2hours	Natsayi
Able to understand is simple terms what a protocol is		
Able to understand why the protocol is so important in research		
Able to know when and how do we use the protocol		
**STUDY STANDARD OPERATIONAL PROCEDURE - SOP**	3hours	Nonhlanhla/ Sithembile
Able to understand what a standard operating procedure is		
Able to understand all SOP’s used by the project		
Able to use the standard operating procedure		

**UNDERSTANDING HIV PREVENTION INTERVENTIONS**	2hours	Nonhlanhla/Natsayi
Able to identify the various HIV prevention interventions available in their community and in general		
Able to identify organisations providing care and treatment in their communities		
Able to identify and refer young people to organisations for support, care and treatment		
**KNOWLEDGE OF RIGHTS OF YOUNG PEOPLE**	1hour	Nonhlanhla
Able to identify the rights of young people including in accessing health, social welfare – right to have an Identification, rights to education and right to accurate and correct information etc		
**BASIC RESEARCH METHODS**	3hours	Natsayi /Nonhlanhla
Able to explain in simple terms why research is important		
Able to list the various methods used to collect data		
Able to list the various tools used to collect data		
Able to explain the uses of data		
Able to explain why data collected has to be accurate		
**Lay counselling**	14days	Outsourced
**Basic HIV/AIDS and other STIs management**	Inclusive in the 14 days	Outsourced
OPTIONS CONSORTIUM (WRHI: Ambassadors training for Peer Navigators)	3days	External/outsourced/ WRHI
**HIV Prevention Ambassador Training**: The HIV Prevention Ambassadors training package was developed to support the meaningful engagement of young people and their communities in the rollout of oral PrEP for HIV prevention.		
