# Supplementary material for: Thetha Nami: participatory development of a peer-navigator intervention to deliver biosocial HIV prevention for adolescents and youth in rural South Africa
Source: BMC Public Health. 2021 Jul 13;21:1393. doi: 10.1186/s12889-021-11399-z (PMC8278686; doi:10.1186/s12889-021-11399-z)
Supplement: Supplementary file 2 — Additional file 2 Thetha Nami Participatory Mapping Workshop Tools. [file 12889_2021_11399_MOESM2_ESM.pdf]

THETHA NAMI TRAINING AND PARTICIPATORY MAPPING TOOLS

Training and workshop dates

Training/Workshop	Training/Workshop Objectives	Training/Workshop Date(s)
Participatory Workshop One	To iteratively co-create and contextually adapt a biosocial peer-led intervention to support HIV prevention. We synthesised quantitative and qualitative data collected between2016 and 2018 into vignettes illustrating the local drivers of HIV. The peer-navigators discussed the vignettes in small groups (nine groups), brainstormed solutions and mapped the components to their own izigodi (local arears).	14/06/2018
Participatory Workshop Two	Community mapping to quickly gain a broad understanding of the social context for adolescents and young people and the reach and coverage of health care services and other non-health services important to young people.	15/11/2018
Participatory Workshop Three	To strengthen a peer led Multi-level HIV prevention intervention. The training sessions provided lessons learned and an opportunity to expand and explore intervention aspects from the theory of change drawn for this intervention.	17/09/2019

Participatory Workshop One – Discussions, Tools and/or Materials

Group one

LOCATING YOUNG PEOPLE FOR A PEER LED INTERVENTION

You are part of a group of potential peer navigators who will participate in a peer led intervention which aims to assist young people to overcome barriers to uptake and retention in multilevel HIV prevention and sexual and reproductive health care. As a peer navigator:

Discussion Points

- How will you find young people in your community?
- How can you define a young person & what would you consider is important to young people?
- How will you make yourself and the work that you do as a peer navigator known by young people in your community?
- How will you identify young people needing HIV and sexual and reproductive health services in your community?
- What resources do you think you will need to do this work?

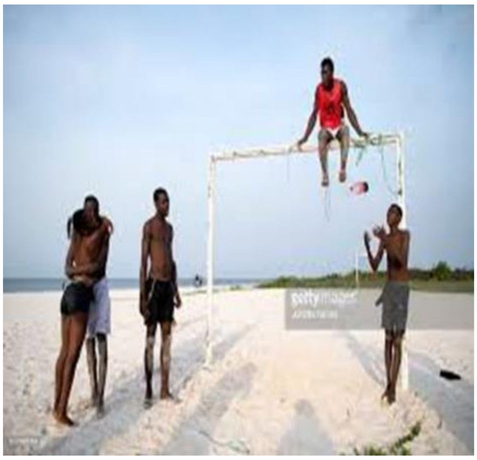

Group two

FINDING HEALTH CARE IN A NEW LOCATION

Buhle – is a 15-year-old girl, she has just moved into your community and she needs HIV and sexual and reproductive health care services. She needs antiretroviral treatment and she also needs contraceptives. Buhle does not know where the services are, and she cannot talk to her aunt and older cousins whom she is staying with.

Discussion Points

- As a peer navigator, how can you help and support Buhle?
- What potential barriers is Buhle likely to come across?
- What possible solutions would you put in place for these barriers?

Group three

Involvement of young men in interventions and care – exposure, awareness, and uptake

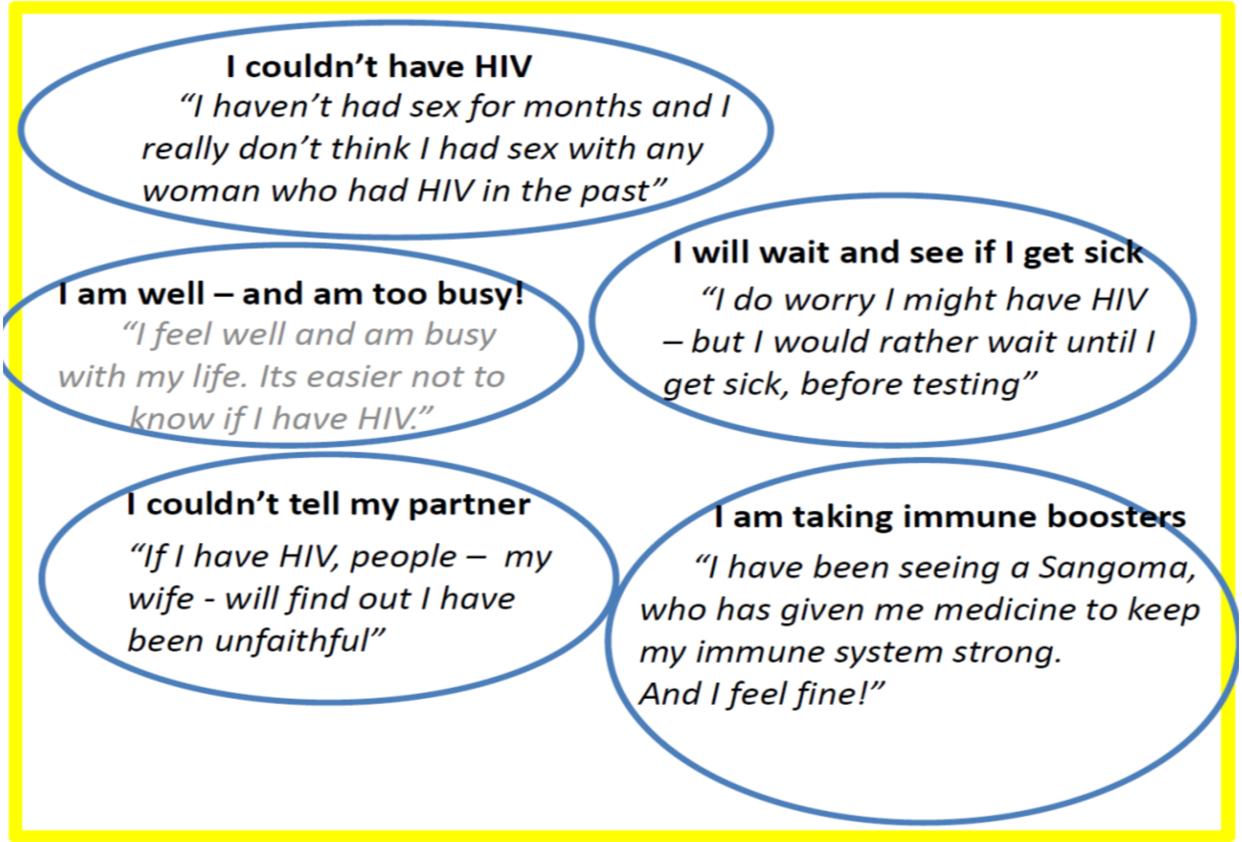

Discussion Points

- Why do you think some young men think/feel this way?
- Some men are less likely to test for HIV and some are not available at home to test for HIV. Why is this so?
- What could make young/older men test for HIV and where would they like to test for HIV?
- Would they like to test themselves using the HIV self-screening kit, if Yes/No, why?
- How can men link to care if tested positive?
- How can HIV+ men be retained in care and stay on ARVS?
- How can we ensure that those who tested negative remain negative?
- What approach/es would work for men to test and stay on treatment?
- What can you as a peer navigator do to help young men in your community to access HIV related care?
- What can you do to help young men to overcome barriers to health care?
- What challenges do you think you will face and what solutions will you put in place?

Group four

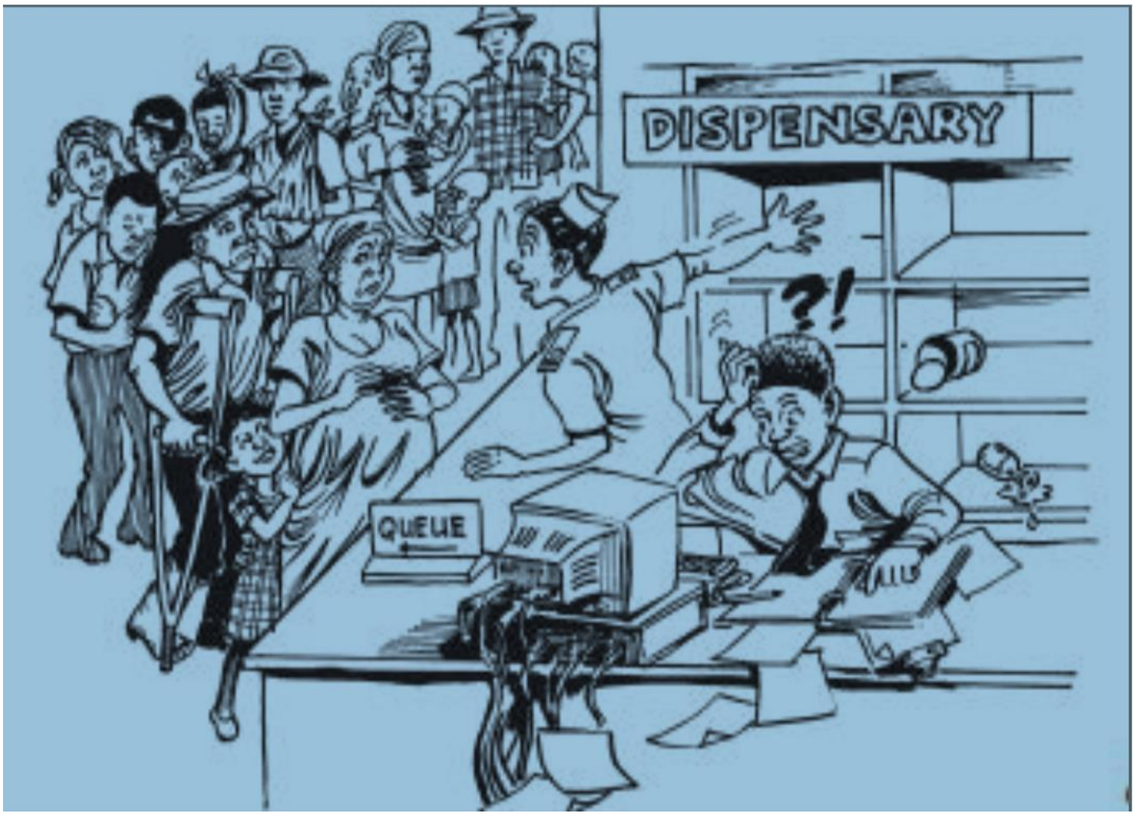

Source: Loewenson et. al, 2006

Discussion Points

- When you look at this picture, discuss what comes to your mind
- When your peers come across this situation in your community, what do they do?

(During discussion, ask peer navigators to think about challenges and solutions, how they fit in and how they can help their peers).

Group five

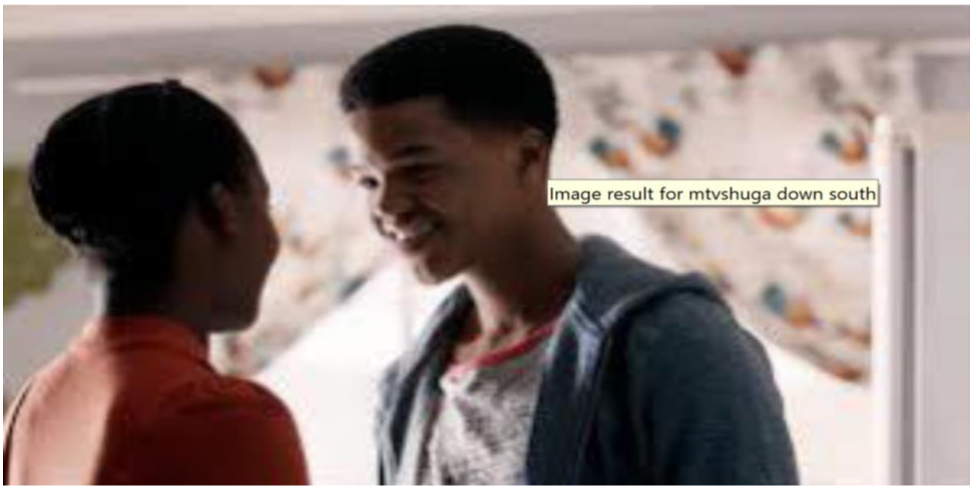

My name is Dumi
I used to think: what if I test positive? I thought that would mean no sex – and no babies, no more doing the things that I like such as sports and no more friends- ever!
I had it all so wrong! It was really an old neighbour that persuaded me to test – he has always been like an uncle to me. He said its Ok to use treatment from a sangoma (traditional health practitioner), but it won't help if its HIV. Go and get tested - then you can protect your girlfriend and your babies from getting infected. But I said – how can I have babies if I must use condoms? And he laughed at me! He said you and your friends have to understand the treatment will stop you being infectious. If you take it very carefully and get your blood test done to make sure it's working – you can't give it to your girlfriend. So – condoms until you agree you want a baby! And if the mother of any future baby did have HIV, she can also protect the baby with the medication. Anyway, he said: get yourself sorted first!

Discussion Points

- Does treatment help to reduce HIV in the body to an extent that you cannot infect your partner and can thus be able to have sex without a condom and have babies? Why?
- What do you know about EMTCT and how do you think you can communicate this information to your peers?
- How can you as a peer navigator communicate HIV related information to your peers? What kind of assistance would you need to communicate this information effectively?
- What possible challenges do you think you are going to face as a young person when talking about HIV and sexual and reproductive health issues with your peers?
- What possible solutions would you put in place to overcome these challenges?

Group six

SUPPORTING YOUNG PEOPLE IN YOUR COMMUNITY

Zodumo completed Matric two years ago, she is 20 years old. Her family does not yet have money to send her to a tertiary institution, so she is staying with her grandmother, two older brothers and a younger sister. Her mother comes home once a month as she works in Johannesburg. Zodumo has been in a relationship for the past three years. Her boyfriend is supportive, he gives her money to buy toiletries, do her hair and buy a few clothing items and she appreciates this gesture. Zodumo loves her boyfriend and feels protected, he is only six years older than Zodumo. Her boyfriend works in Durban and he comes to the local area on weekends. When he comes, he expects Zodumo to visit him and they have been sexually involved since the beginning of their relationship. During the first six months, they used condoms, but her boyfriend convinced her that their love was growing, and they should stop using condoms then Zodumo started using an injection to prevent pregnancy, she finds the nurses at the clinic friendly and she goes to the clinic during "happy hour" so she only spends less than two hours and goes home. Zodumo and her boyfriend have never tested for HIV, because Zodumo's boyfriend told her that he is HIV negative, and Zodumo has never had a sexual relationship with anyone else so she also knows she is negative. Zodumo knows that her boyfriend has a "baby mama" in Durban but he told her that they are no longer together.

Discussion Points

- What advice would you give to Zodumo about her relationship?
- Define risk and protective factors for Zodumo
- What solutions would you have for Zodumo's risks?
- What challenges do you think you will face as a peer navigator in trying to help Zodumo and how you will overcome those challenges?

Group seven

BASKET OF HEALTH SERVICES IN YOUR COMMUNITY

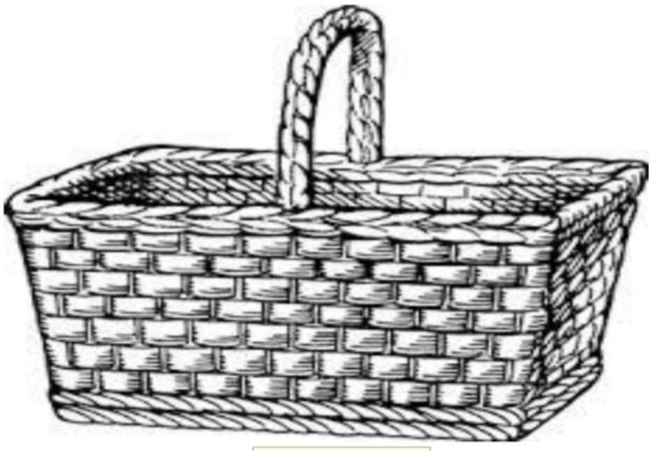

Discussion Points

- List all HIV and sexual and reproductive health-related services in your community
- Which ones are easily accessible/difficult to access and why?
- What can you as a peer navigator do to help improve access to services that are not easily accessible?
- Which services are related/not related? Use a Venn diagram to examine the relationships between services (as per young people's perceptions). Also examine preferences for use of services
- Are there other alternative services used by young people? For what? How are they accessed?

Group eight

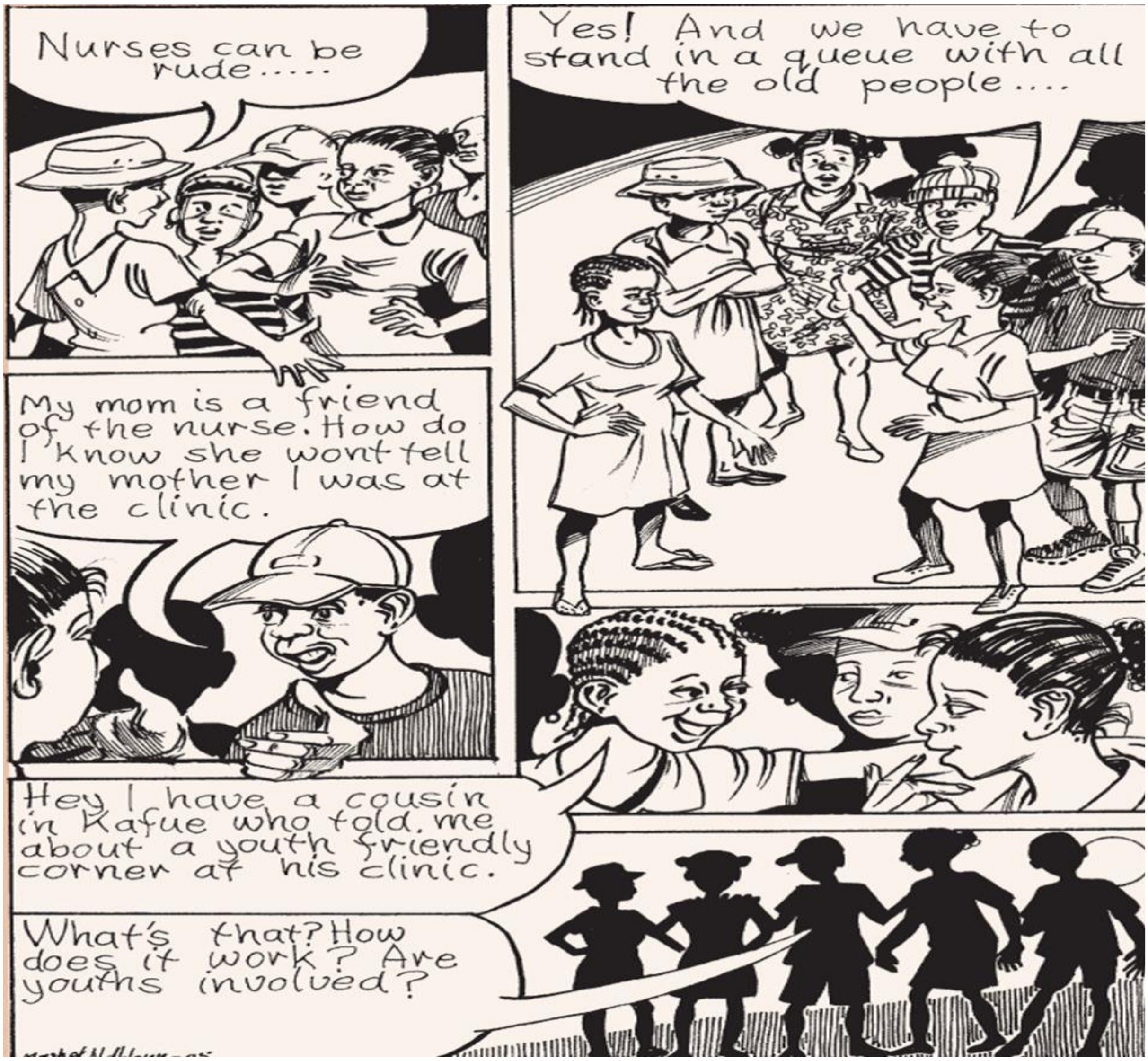

Source: Loewenson et.al, 2006

Discussion Points
"They (nurses) are full of themselves; they are not afraid to take a lunch break for an hour. When you try and talk, you are told they are on lunch. You will get there and stay and by the time when they get out it is almost the time to leave. You find that sometimes they will take lunch breaks at 11 or 12 until" (from a FGD with young men)

- Why do young people find it difficult to access health care?
- List barriers and facilitators to health care…..ranking them from those affecting young people the most…to the least
- What can you as a peer navigator do to help young people to overcome these barriers?

Group nine

HIV PREVENTION AND REPRODUCTIVE HEALTH

Interview extract

INTERVIEWER: What do you understand about abstaining?
PARTICIPANT: In my view it means not engaging in sexual intercourse.
INTERVIEWER: What other methods of prevention, do you know about?
PARTICIPANT: It is for a girl to drink a mixture of coke and disprin after having a sexual intercourse, so that the sperms will not penetrate through, but remain on the surface.
INTERVIEWER: Does that prevent transmission of Sexually Transmitted Infections, what else does it prevent?
PARTICIPANT: It also prevents pregnancy, but mainly the transmission of HIV.
INTERVIEWER: What other methods of prevention do we know about?
PARTICIPATN2: Another method is consuming the seed of dagga; the girl drinks the seed of dagga and it becomes a permanent contraceptive.
INTERVIEWER: How is this taken?
PARTICIPANT2: It is taken as you would be drinking a cold drink or water, it is said when taken, it goes and blocks something in the uterus.
INTERVIEWER: Is it taken as is, just like that?
PARTICIPANT: Yes, it is taken just like that.
INTERVIEWER: What is the egg of dagga?
PARTICIPANT: It is the seed of dagga.

Discussion Points

- Discuss groups' insights about the above extract
- Discuss these type-HIV prevention and reproductive health methods used by young people
- What are the roles of peer navigators in creating and dispelling myths about HIV and reproductive health?
- What do peer navigators think can help to dispel myths and to communicate factual/correct information to their peers?

Participatory Workshop Two – Discussions, Tools and/or Materials

Peer navigators were trained in community mapping with a focus on theoretically assisting them on the following activities:

- How they would gain a broad understanding of the communities where they would be implementing their work
- How they would liaise with different structures, including religious systems, families, civil society, government departments, and traditional authority
- How they would create and sustain safe spaces for young people
- How they would debrief, monitor and record their work in the field.

- **Community Mapping**

Components of community mapping highlighted above were discussed in detail, allowing peers to ask questions for clarity and think about how they would go about conducting the above activities during the pilot, in preparation for implementation of their actual work.

- **Liaising with different structures**

Peers reported that they would use diverse platforms to liaise with different structures. Some of the platforms mentioned included youth groups in churches, approaching young people in their households and where they hung out, involving churches by approaching different congregations, liaising with civil society organisations in different communities, using already existing structures such as community care givers (CCGs) to access different government departments, attending traditional authority meetings in their communities in order to present themselves and get to understand youth issues discussed in these gatherings.

Possible barriers:

- Fear of the possibility of being taken for granted and not trusted by young people and other community members
- Getting influential people in the community on their side. Influential people included politicians and business owners. They said that these were influential stakeholders to have on their side, however, most of them were not interested on issues that concerned young people.

Discussion Points:

- Build a map of your own community
- Identify barriers and facilitators of working with the different structures
- Who has a lot of power but is not interested in community/youth affairs?
- Who has interest but no/less power in community/youth affairs?
- Which stakeholders have power and interest?
- Which structures do young people have interest in?

- **Creating safe spaces**

Peers thought about buildings as safe spaces. They were reminded during training that safe spaces did not only include buildings, but they had to also consider emotional, physical and psychological

safety of the spaces they were going to create for young people. For the emotional and psychological elements, young people had to know that if they needed such support, it was going to be provided to them privately (if they chose). Peers mentioned that they would use social media platforms to engage young people about safe spaces, particularly with regards to advertising these spaces. Proposed platforms included WattsApp groups, Facebook, Twitter. If these could be feasible, a discussion on phones and/or data was initiated.

They also mentioned that they would use recreational activities such as sports, role plays, music and MTV SHUGA screening to engage young people.

It was noted that it could be difficult to retain young people in safe spaces, however, peers thought that, apart from discussing health related issues (and other issues of concern to young people) if they brought activities such as music, screening SHUGA, and sports, young people could stay engaged.

Venues considered for safe spaces were community halls, with the possibility of exploring using school halls, tuck-shops, back rooms.

They were also asked to think about whether their safe spaces would be in fixed venues, at fixed times or whether they would conduct them in different venues and different times. This needed further exploration which would be done during community mapping.

Peers were also asked to think about how they would record safe space activities. They mentioned taking notes, photos and perhaps using tablets to type out the notes and send them to the office for filing.

Discussion points:

- In your community, which spaces do you think will be safe for young people?
- How will you practically create a safe space for young people in your community?
- What do you think will work/won’t work and how will you sustain it?
- What do you think you will need to create this safe space?
- How will you help young people to access the safe space within their community?
- How will you advertise your activities?
- How will you use the safe spaces to communicate SRH, U=U, TasP, UTT, self-testing, PreP, HCT, alcohol, mental health messaging? Including other non-health activities/issues for young people
- What kind of community delivery models you think will work in your community?
- How will you use the safe space to communicate and deliver health interventions and which ones?

Participatory Workshop Three – Discussions, Tools and/or Materials

The third workshop included two sections. In the first section, peer navigators identified their strengths and highlighted challenges and barriers for conducting Thetha Nami. In the second section, they were divided into three groups to discuss how they would overcome the barriers identified.

Activity 1: Identifying Challenges and strengths:

The third training workshop was divided into two sections. The first section (which took place from 9h00 to 11h00) was conducted to explore peer navigators' experiences of doing a health promotion intervention from March-September 2019 – 6 months.
In the first section, peer navigators identified challenges and strengths of Thetha Nami in their communities.

Once the group had engaged in discussions about their challenges/strengths, a ranking exercise followed to distinguish issues which peer navigators thought needed immediate attention and those which needed attention as the intervention continues.

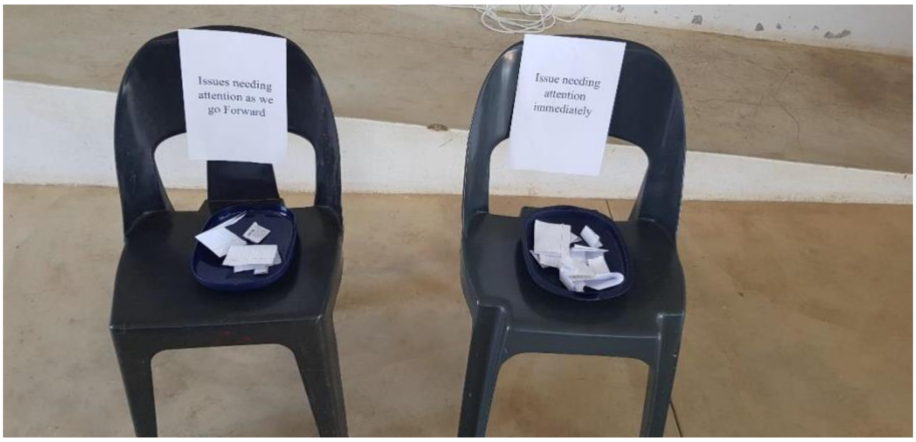

Ranking exercise: issues needing immediate attention and issues needing attention as the intervention goes forward

At the end of the ranking exercise, peer navigator's votes were counted

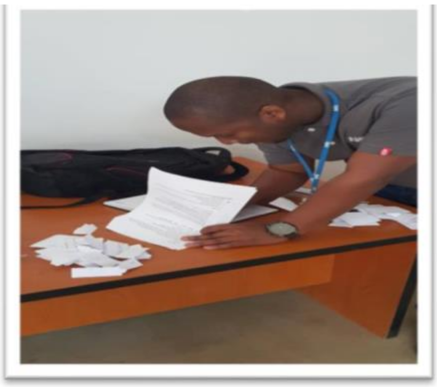

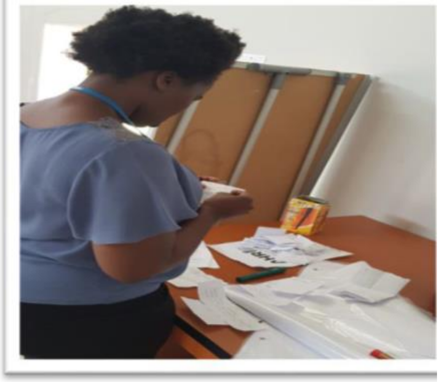

Activity two:

The second part of the training workshop was scheduled for two hours and a third hour was used to have a discussion/feedback session with all peer navigators.

In this section. Three issues were selected (the first three issues identified from the ranking exercise) for an in-depth discussion. Issues selected for discussion were based on the votes received. The aim of this second session was to start initial discussions around issues identified by peer navigators so that ways to deal/solve issues could be implemented once peer navigators have a step to step - how to?
Peer navigators were divided into three groups and each group had a facilitator and note taker. Discussions were recorded.
