# Supplementary material for: Thetha Nami: participatory development of a peer-navigator intervention to deliver biosocial HIV prevention for adolescents and youth in rural South Africa
Source: BMC Public Health. 2021 Jul 13;21:1393. doi: 10.1186/s12889-021-11399-z (PMC8278686; doi:10.1186/s12889-021-11399-z)
Supplement: Supplementary file 3 — Additional file 3 Thetha Nami Peer Navigator Monitoring Tool. [file 12889_2021_11399_MOESM3_ESM.pdf]

Multilevel HIV Prevention Study: Using family and peer support to improve uptake and retention in HIV prevention for adolescent girls and young women

Peer navigator Monitoring tool

PARTICIPANT DEMOGRAPHIC INFORMATION/SURVEY

Peer navigator's Full Name: ____

Date: ______________ Time: _____________________

Location/Izigodi: ______________________

Interviewee's Full Name: ____________________________ Izigodi: _______________

Occupation: ______________________________ Participants' Mobile telephone #: _______________________

Participants' alternative mobile telephone #___________________

Age [in years]: _____________ ID Number: ______________________________

DSID___________________________ BSID_________________________________

Sex [circle one]: FEMALE MALE Marital Status: SINGLE MARRIED IN A RELATIONSHIP

1. Place where young person was identified:
   School
   Health care facility – clinic/hospital
   Home
   Town
   Watering place
   Social gathering specify …………………
   Sporting event specify……………………
   Community meeting………………………………
   Other specify……………………………………

2. Type of need the young person has:
   Health Y/N don't know
   Social eg need grants, employment advice, Y/N/DK
   Social vulnerability e.g. violence, psychosocial, child protection, alcohol/substance addition etc Y/N DK (if yes then please discuss with supervisor when making plans)
   Education Y/N/DK
   Legal Y/N/DK
   Other specify………………………………

DURBAN
K-RITH Tower Building, 719 Umbilo Road, Durban
Private Bag X7, Congella, 4013, South Africa
T +27 (0)31 260 4991
E durban@ahri.org

SOMKHELE
Africa Centre Building, via R618 to Hlabisa, Somkhele, Mtubatuba
PO Box 198, Mtubatuba, 3935, South Africa
T +27 (0)35 5507500
E somkhele@ahri.org

www.ahri.org

Africa Health Research Institute (AHRI)
is the operational name of K-RITH (NPC),
a Registered Non-Profit Company 2011/011985/08

Please list and specify the type of assistance they required from you eg knowledge of where to get contraception, help with getting an ID book, assistance with educational bursaries…………………………………………………………………………….……

Tick any that apply

3. HIV care and prevention information and counselling Yes/No
4. Sexual and reproductive health Yes/No
5. Safe spaces Yes/No
6. Mentor program (age 15-24) Yes/No
7. Social Assets programs - Financial capabilities and Vhutsilo (age15 24)
8. Gender norms and Violence prevention related programs - eg. Stepping Stones and Violence prevention education and Gender norms-related education (talks or activities), eg. Vhutshilo 1&2 Yes/No
9. Financial literacy training for girls/young women and young boys/men, eg. ASPIRES, Vhutshilo and Financial Capabilities and Savings group incl local program to promote savings, like Stockvel and Microfinance programs (Financial services incl loans, savings insurance available to poor entrepreneurs and SME who have no collateral to apply for a loan), run savings clubs and opening bank accounts. Yes/No
10. Vocational skills training, Business/entrepreneurial training Yes/No
11. Local program for caregiving, e.g. Parent/care-giver positive caregiving program, or programs for parents about improving adolescent-parent communication e.g.Teenagers and Adults Learning to Communicate (TALC) (Let's Talk!) Yes/No
12. HIV education in school or Life skills-based education / Life orientation program curriculum used in school Yes/No
13. HIV testing programs available. Have you ever heard or been part of such programs? Yes/No
14. Male circumcision (voluntary medical circumcision) (Not traditional circumcision) Yes/No
15. Condom promotion or provision Yes/No
16. SRH services Yes/No
17. Health services Yes/No
18. Adolescent-friendly services like happy hour, priority queues, etc) Yes/No
19. Post-violence care, (including sexual, physical or emotional): Yes/No
20. Legal, Linkage to judiciary, Police, Affidavit) Yes/No
21. Cash transfer and support for school fees, uniform, books or stationery Yes/No
22. CV writing/ career support Yes/No
23. Education support Yes/No
24. Recreation Yes/No

Did the young person require any referral? Yes/No

If yes, can you please indicate where and what kind of referral services they needed, and the action taken?.......................................

Where/ from which organisation provided it? [repeat this question for all of the following programs] tick all the apply:

DoH
Private Doctor
Pharmacy
Africa Centre / AHRI
FHI 360
Humana People to People South Africa (HPPSA)
Hope International
NACOSA
EDI
Ezibayeni

Unyezi
Isikondlakondla
MatCH
FPD
AFSA
TB/HIV Care Association
DoE
Mpilonhle
Star for Life
Other specify …….
I don't know

25. Is the young person currently in/out of school?
    In school
    Out of school
    Completed matric
26. If in school – what grade is the young person? ……………
27. Are they repeating a grade or upgrading their subjects? Yes/No
28. Has the young person recently moved into the area or they have always been resident?
    Recently moved in
    Always resident
29. Is the young person employed or not? Employed/Not employed/Self employed/Employed Part time/Employed full time

**Your view of the young person you met**	**A lot** 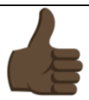	**A little** 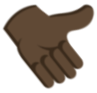	**No** 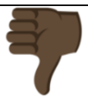
**Easy to talk to**			
**Keen to listen to your messages**			
**Worried about the subjects I raised**			
**Seemed very isolated with no one else to talk to**			
**Seemed very popular with lots of other people to talk to**			
**Will follow your aadvice?**			
