# Supplementary material for: Thetha Nami: participatory development of a peer-navigator intervention to deliver biosocial HIV prevention for adolescents and youth in rural South Africa
Source: BMC Public Health. 2021 Jul 13;21:1393. doi: 10.1186/s12889-021-11399-z (PMC8278686; doi:10.1186/s12889-021-11399-z)
Supplement: Supplementary file 4 — Additional file 4. Peer Navigator indepth interview topic guide. [file 12889_2021_11399_MOESM4_ESM.pdf]

Multilevel HIV Prevention Study: Using family and peer support to improve uptake and retention in HIV prevention for adolescent girls and young women

Topic guide: interviews with peer navigators

Time: 30 minutes -1 hour

Topic guide to explore young people' experiences, expectations, and perceptions of health care (sexual and reproductive health, and HIV treatment and prevention services and other health care and wellbeing interventions) and the peer navigator intervention

Peer navigator Study ID#: ____

Date: ______________ Time: _____________________

Location/Izigodi: ______________________

Age [in years]: _____________

Sex [circle one]: FEMALE MALE

A. Health care needs, experiences and expectations

- What are the general health care needs of young people in your community? What have their experience been like?
- What were the good things? What were the bad things? What could be better?
- What health care (and wellbeing) needs do young people and yourself need? (ranging from health, social, legal, psychological, educational and other needs)

Please describe/explain the type of assistance they required from you eg knowledge of where to get contraception, help with getting an ID book, assistance with educational bursaries

B. Experience and expectation of HIV testing and sexual and reproductive health

- Do young people know where to test?
- Have they tested and how was the experience?
- If they haven't tested, explore the reasons not and what sorts of things would make it easier to test? What did they say was their experience HIV care – both for positive and negative?
- What do they understand about fertility and what impacts on it?
- What do they envisage as safe pregnancy?
- Ask where they received their information around fertility and sex and what do they feel about the way in which they were taught about it? Who did they receive the information from?

C. Perceptions and expectations about the peer navigator intervention

- How important do you think it is to have peer navigators in your community?
- Do young people think peer navigators help or not? Probe reasons why?
- Do they see any change in their community (with the presence of navigators) or not? Please provide reasons for response
- What do young people expect peer navigators to do?
- Can you please describe your experience?
- Please describe the activities the peer navigators were involved in? What were you involved in? How was your experience?

D. Mobility

- Where young people gather with others and what they do in these places?
