# Supplementary material for: Thetha Nami: participatory development of a peer-navigator intervention to deliver biosocial HIV prevention for adolescents and youth in rural South Africa
Source: BMC Public Health. 2021 Jul 13;21:1393. doi: 10.1186/s12889-021-11399-z (PMC8278686; doi:10.1186/s12889-021-11399-z)
Supplement: Supplementary file 5 — Additional file 5 Thetha Nami Peer Mentorship Tool. [file 12889_2021_11399_MOESM5_ESM.pdf]

Isisekelo Sempilo: HIV prevention embedded in sexual health: A pilot trial to optimize Thetha nami (peer-delivery) of HIV prevention and care to adolescents and young adults in rural KwaZulu-Natal

Thetha Nami Follow Up

Contact Attempts

Individual Record ID (Auto Number)

Contact Attempts (up to 6 attempts)

Participant's Contact Information

BSID
Name of the Bounded Structure Owner
Name of the Household head
Participant's Surname
Participant's First names
Participant's Sex
Participant's Age
Week block
Week
Participant's Primary Contact Number
In which Study Arm:
Staff Member Code:

Attempt Date and Time………………………………

Was the participant contacted No/Yes, Yes, but participant is mentally incapacitated

Was the participant willing to be surveyed/assisted/followed-up? No/Yes

Reschedule next visit date……………………….

Refused participation……………………..

Who refused participation? Self; Partner; Household Head; Bounded Structure Owner; Parent/Guardian; Other

Specify other person who refused participation……………………………………

FOR FIELD TEAM

Non-Contact - Handover to Project Coordinator

Fieldworker: Request handover of Non-Contact to Project Coordinator? No/Yes

Select reason for non-contact in field

Not found after 4 attempts
Temporarily away for more than 2 months Stay in currently Avoided BS/HH
Out-migrated to unknown destination
Out-migrated within PIPSA, but unable to visit Contacted, but unable to conduct survey after 4x contact attempts
Unable to acquire parental consent after 4x contact visits
Reported to be involved in gang activities Other
Specify other reason for non-contact from field:,………………………………………

Fieldworker: Please provide narrative

Premature completion

Was this a Pre-mature completion? No/Yes

Select reason for pre-mature completion Reported dead? Out migrated outside PIPSA, Other

Specify other reason for pre-mature completion:…………………………………

Visit Information: Visit Date………………………………………

Personal Information

Individual Record ID (Auto Number)
Ulwazi lomuntu siqu Personal Information
Actual BSID
Coordinate - Latitude Coordinate - Latitude
Coordinate - Longitude Coordinate - Longitude
Isibongo Surname
Igama Firstname/s
Sicela unikeze usuku lokuzalwa okuyilona Please provide the correct Date of Birth…………………….
Inombolo kamazisi ebhaliwe noma inombolo yesitifiketi sokuzalwa Pre-printed National ID or Birth certificate number……………………………..
Ubulili Sex Male/Female
Sicela ucacise inombolo kamakhala ekhukhwini ongathanda ukufonelwa kuyo Please provide your primary contact number (mobile)…………………………………………..

Location/Izigodi:

Ebaswazini
Esiyembeni
Gunjaneni
Kwamsane Reserve
KwaMsane Township North
KwaMsane Township South
Kwahoho

Machibini
Macambini
Mahunjini
Mapheleni
Mshaya
Myeki
Mvutshini
Indlovu Village
Nkolokotho
Nkombose North
Nkombose South
Nomathiya
Nqopheni
Nsolweni
Ophondweni
Ogengele
Ophaphasi

Introduction peer navigator name and institution and name of study:

Hello I am ……………… from AHRI and I am working as a Thetha Nami peer navigator in the HIV prevention programme.

Memory Note

- Greet the participant with respect irrespective of their age
- Ask if they can give you few minutes of their time.
- If yes, introduce yourself and the organization
- Introduce Theta Nami and the Isisekelo sempilo study and make you sure you mention that we are trying to understand young people's need and how we can support them.
- Remember to capture their needs on the tablet-based monitoring tool.
- If requested, please provide support and refer participant to relevant services (check the list of services with you).

Is the person willing to speak to you? No/Yes

What is the main reason why this participant is not willing to speak to you? Not interested; In a hurry, can't speak now; Other

Please provide other reason why the person was not willing to speak to you………………………….

Goal of Thetha Nami

The goal of the Thetha Nami is to help young people aged 16-30 years to navigate their communities, support and link them into HIV prevention, treatment and care services among social and education issues you may have.

Thetha Nami Survey

Place where young person was found:

School
Health care facility - clinic/hospital Home
Town

Watering place
Social gathering specify Sporting event specify Community meeting Other specify
Please provide other/more details regarding the place where this person was found:…………….

Type of need the young person has: Yes No Don't know

Health
Social e.g. need grants, employment advice
Social vulnerability e.g. violence, psychosocial, child protection, alcohol/substance addition
Education Legal
Other, specify below
Please specify details regarding the other type of need……………………………………

Please list and specify the type of assistance they required from you eg knowledge of where to get contraception, help with getting an ID book, assistance with educational bursaries

HIV testing, care and prevention information and counselling Tick any that apply [repeat responses for all services below]

Information provided
Referral
Not needed
N/A

To which organisation/s was the person referred to? [repeat for all services below]

Isisekelo Sempilo Clinic
DoH
Private Doctor CCG
EDI Isikondlakondla
DsD
Ezibayeni Unyezi Isikondlakondla
TB/HIV Care Association DoE
Mpilonhle Star for Life
DsD
SAPS
Youth Champion CCG
Tribal Authority Municipality
I don't know
Other specify ....
Please provide details about another Provider………………………………….

PrEP counselling

Male circumcision (voluntary medical circumcision) not traditional circumcision

Condom promotion or provision

Adolescent-friendly services

Sexual and reproductive health

Safe spaces

Social Assets programs - Financial capabilities

Gender norms and Violence prevention related programs - e.g. Stepping Stones and Violence prevention education and Gender norms-related education (talks or activities), e.g. Vhutshilo

Financial literacy training for girls/young women and young boys/men, e.g. Stokvel and Microfinance programs (Financial services including loans, savings insurance available to poor entrepreneurs and SME who have no collateral to apply for a loan), run savings clubs and opening bank accounts

Vocational skills training, Business/entrepreneurial training

Local program for caregiving, e.g. Parent/care-giver positive caregiving program, or programs for parents about improving adolescent-parent communication

HIV education in school or Life skills-based education/Life orientation program curriculum used in school

Post-violence care, (including sexual, physical or emotional)

Legal, (Linkage to judiciary, Police, Affidavit)

CV writing/career support

Education support

Recreation
